# How Is the Fidelity of Proteins Ensured in Terms of Both Quality and Quantity at the Endoplasmic Reticulum? Mechanistic Insights into E3 Ubiquitin Ligases

**DOI:** 10.3390/ijms22042078

**Published:** 2021-02-19

**Authors:** Ji An Kang, Young Joo Jeon

**Affiliations:** 1 Department of Biochemistry, College of Medicine, Chungnam National University, Daejeon 35015, Korea; wldksdl555@naver.com; 2 Department of Medical Science, College of Medicine, Chungnam National University, Daejeon 35015, Korea

**Keywords:** endoplasmic reticulum (ER), ER-associated degradation (ERAD), ER-phagy, E3 ubiquitin ligase, protein quality control, protein quantity control, ubiquitin

## Abstract

The endoplasmic reticulum (ER) is an interconnected organelle that plays fundamental roles in the biosynthesis, folding, stabilization, maturation, and trafficking of secretory and transmembrane proteins. It is the largest organelle and critically modulates nearly all aspects of life. Therefore, in the endoplasmic reticulum, an enormous investment of resources, including chaperones and protein folding facilitators, is dedicated to adequate protein maturation and delivery to final destinations. Unfortunately, the folding and assembly of proteins can be quite error-prone, which leads to the generation of misfolded proteins. Notably, protein homeostasis, referred to as proteostasis, is constantly exposed to danger by flows of misfolded proteins and subsequent protein aggregates. To maintain proteostasis, the ER triages and eliminates terminally misfolded proteins by delivering substrates to the ubiquitin–proteasome system (UPS) or to the lysosome, which is termed ER-associated degradation (ERAD) or ER-phagy, respectively. ERAD not only eliminates misfolded or unassembled proteins via protein quality control but also fine-tunes correctly folded proteins via protein quantity control. Intriguingly, the diversity and distinctive nature of E3 ubiquitin ligases determine efficiency, complexity, and specificity of ubiquitination during ERAD. ER-phagy utilizes the core autophagy machinery and eliminates ERAD-resistant misfolded proteins. Here, we conceptually outline not only ubiquitination machinery but also catalytic mechanisms of E3 ubiquitin ligases. Further, we discuss the mechanistic insights into E3 ubiquitin ligases involved in the two guardian pathways in the ER, ERAD and ER-phagy. Finally, we provide the molecular mechanisms by which ERAD and ER-phagy conduct not only protein quality control but also protein quantity control to ensure proteostasis and subsequent organismal homeostasis.

## 1. Introduction

Post-translational modification (PTM) by ubiquitin (ubiquitination) is a very powerful and finely tuned process that is spatiotemporally involved in nearly all aspects of biological and physiological functions for the maintenance of organismal homeostasis. Comprehensive proteomics studies have identified tens of thousands of ubiquitination sites on thousands of proteins, indicating that the vast majority of cellular proteins are ubiquitinated during their lifetime. Among the enzymes catalyzing ubiquitination—including E1 ubiquitin-activating enzymes, E2 ubiquitin-conjugating enzymes, and E3 ubiquitin ligases—E3 ubiquitin ligases govern the efficiency, complexity, and specificity of ubiquitination, since E3 ubiquitin ligases participate in the specific recognition of signals present on substrate proteins [1,2,3,4,5,6,7]. Importantly, ubiquitination leads to alterations in activity, interaction, intracellular trafficking, stability, and subcellular localization of substrate proteins through its reversible nature, rapid kinetics and versatility of outcomes, thereby orchestrating a plethora of biological processes involving stress responses, immune modulation, signaling transduction, control of cell cycle, division, and proliferation [5,8,9,10,11,12,13,14,15]. Therefore, aberrant function or dysregulation of ubiquitination could be at the roots of the development of many severe diseases such as cancer, immune disorders, neurodegeneration and susceptibility to infections.

In the endoplasmic reticulum (ER), folding of proteins is inherently error-prone. Even with an elaborate network to ensure adequate protein folding and assembly, misfolding and subsequent aggregates of proteins can occur quite frequently. To maintain protein homeostasis, referred to as proteostasis in the ER, eukaryotes have evolved multiple quality control mechanisms involving ER-associated degradation (ERAD), ER-phagy, Golgi quality control, and plasma membrane quality control [16]. While most of the misfolded proteins in the ER are efficiently eliminated via ERAD, ERAD can be constrained by aggregation propensity, the severity of mutation, the nature of folding lesions and kinetics, and the thermodynamics of folding, which leads to the development of another mechanism, ER-phagy. Interestingly, some misfolded proteins are subject to more than one quality control, suggesting the cooperation of the quality control mechanisms to efficiently eliminate the misfolded proteins. Preference among ERAD and ER-phagy could rely on the versatile characteristics of substrate proteins, including topology, misfolding positions and modifications of substrate proteins [17].

In this review, we first conceptually outline ubiquitination machinery and catalytic mechanisms of E3 ubiquitin ligases. We then highlight the mechanistic insights into the E3 ubiquitin ligases involved in the protein quality and quantity control processes at the ER. We finally discuss both protein quality control and quantity control at the ER, which are mainly mediated by ERAD and ER-phagy.

## 2. Ubiquitination

### 2.1. A Conceptual Overview of Ubiquitination

Ubiquitin is a small, compact and highly conserved globular protein, with the exception of its unrestrained and flexible C-terminal tail. Ubiquitin harbors a unique functional isoleucine–hydrophobic patch that supports essential noncovalent interactions during ubiquitination and signal recognition [18]. To achieve abundant cellular concentrations of ubiquitin, four different genes, *UBB*, *UBC*, *RPS27*, and *UBA52*, encode ubiquitin in mammals. Genes *UBB* and *UBC*, as polyubiquitin cassettes, encode linear fusions of three and nine ubiquitin molecules, respectively, while *RPS27A* and *UBA52* encode ubiquitin as in-frame fusion to small and large ribosomal proteins, respectively, from which deubiquitinating enzymes (DUBs) cleave off the monomeric ubiquitin [19,20,21].

**Figure 1:**
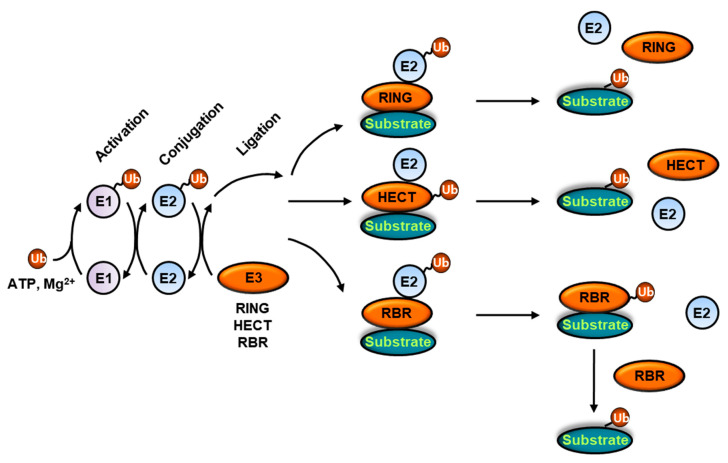
The ubiquitination machinery. Ubiquitination utilizes a three-step enzymatic cascade. Ubiquitin is activated by the E1 ubiquitin-activating enzyme at the expense of ATP and subsequently conjugated to the E1 ubiquitin-activating enzyme via thioester bond. The E1 ubiquitin-activating enzyme then associates with the E2 ubiquitin-conjugating enzyme and transfers ubiquitin from its catalytic cysteine to the active-site cysteine of the E2 ubiquitin-conjugating enzyme to form a thioester bond. Depending on the E3 ubiquitin ligase involved, ubiquitin linked to the E2 ubiquitin-conjugating enzyme can be transferred and subsequently conjugated to the substrate protein via at least two mechanisms. Homologous to the E6-associated protein (E6AP) carboxyl terminus (HECT)-type and really interesting new gene (RING)-in-between-RINGs (IBR)-RING (RBR)-type E3 ubiquitin ligases mediate ubiquitin transfer to the substrate proteins via a two-step reaction, in which ubiquitin is transferred to an active-site cysteine on the E3 ligase, resulting in the formation of a thioester with ubiquitin, and then to the substrate. RING-type E3 ligases catalyze a direct transfer of ubiquitin from the ubiquitin-charged E2 conjugating enzyme to the substrate proteins.

Ubiquitination is a multistep process achieved by E1 ubiquitin-activating enzymes, E2 ubiquitin-conjugating enzymes and E3 ubiquitin ligase sequentially activating, conjugating and ligating ubiquitin to substrate proteins (Figure 1) [8,22]. E1 ubiquitin-activating enzymes consist of ubiquitin-like modifier activating enzyme 1 (UBA1) and UBA6. E1 activating enzymes possess a multidomain architecture, including the adenylation domain, which binds ubiquitin and ATP-Mg^2+^, the catalytic cysteine domain and the ubiquitin-fold domain (UFD), which supports interactions with E2 conjugating enzymes [23,24,25,26,27]. Additionally, a long flexible linker connects the active adenylation domain with the catalytic cysteine domain [28]. Upon binding to both ubiquitin and ATP-Mg^2+^, the E1 ubiquitin-activating enzyme mediates the formation of a high-energy ubiquitin–AMP complex via ubiquitin C-terminal acyl adenylation and then a catalytic cysteine on the E1 activating enzyme replaces the AMP group in the ubiquitin–AMP complex, thereby leading to the generation of an activated ubiquitin via a covalent thioester bond [29].

The E1 ubiquitin complex transfers ubiquitin to the E2 ubiquitin-conjugating enzyme through a transthioesterification reaction [30]. There are approximately 40 E2 ubiquitin-conjugating enzymes in human genome, of which 35 are dedicated to ubiquitination. E2 conjugating enzymes possess a core ubiquitin conjugation fold (UBC), along with N-terminal and C-terminal extensions [31,32]. In accordance, E2 conjugating enzymes are subdivided into class II, which possess N-terminal extensions, class III, which possess C-terminal extensions, class I, which possess neither extensions, and class IV, which possess both N-terminal and C-terminal extensions [32].

E3 ubiquitin ligases finally associate with both the E2 ubiquitin complex and a substrate protein to catalyze the covalent transfer of ubiquitin, thereby leading to the formation of an amide bond of ubiquitin, most commonly with a lysine residue or the N-terminus of the substrate [33]. Interestingly, ubiquitination of non-lysine residues has been reported [34,35]. Ubiquitin can be targeted to thiol or hydroxyl side chains in cysteine, serine, and threonine residues on the substrate proteins [35,36,37,38]. Moreover, ubiquitin can also be conjugated to a serine residue on the substrate protein in a phosphoribosylation-dependent manner [39,40].

DUBs reverse ubiquitination and switch off the ubiquitin signal, which deals with the matched complexity of ubiquitination. In humans, more than 100 DUBs have been identified. DUBs consist of six structurally distinct families of different cysteine and metalloproteases. The five families of cysteine proteases are the ubiquitin-specific proteases (USPs) with 54 members, the ubiquitin C-terminal hydrolases (UCHs) with four members, the ovarian tumor proteases (OTUs) with 16 members, the Josephin family with four members and the motif interacting with ubiquitin (MIU)-containing novel DUB family (MINDYs) with four members [14,41,42,43]. The sixth family of DUBs is Zn-dependent JAB1/MPN/MOV34 metalloproteases (JAMMs) with 16 members [42]. The biochemistry and the roles of DUBs in regulating diverse cellular processes are well-appreciated in recent reviews and thus we will not describe DUBs in further detail here.

### 2.2. Understanding the Catalytic Mechanisms of E3 Ubiquitin Ligases

Despite the mechanism of catalysis being conserved in all eukaryotes, the network of ubiquitin is more sophisticated in mammals due to the large number of E2 ubiquitin-conjugating and E3 ubiquitin ligase enzymes, compared to only two E1 ubiquitin-activating enzymes. There are approximately 40 E2 conjugating enzymes and 700 E3 ligases in humans that function in numerous combinations to ubiquitinate virtually every protein in the cell [44]. E3 ubiquitin ligases determine the efficiency, complexity, and specificity of ubiquitination, since E3 ligases participate in the specific recognition of signals present on the substrate proteins and E2 ubiquitin-conjugating enzymes work only with a limited set of E3 ligases [1,2,3,4,5,6,7].

E3 ligases are divided into three main families on the basis of the structure of the ubiquitin-charged E2 conjugating enzyme-binding domain and the ubiquitin transfer mechanism (Figure 1) [5,9,33]. Really interesting new gene (RING) E3 ligases are characterized by their RING or U-box catalytic domain and catalyze a direct transfer of ubiquitin from the ubiquitin-charged E2 conjugating enzyme to the substrate proteins. With approximately 700 predicted E3 ligases, the RING E3 ligases constitute the largest family of E3 ligases. RING domains are structurally characterized by the presence of two zinc ions essential for RING domain folding, which are coordinated by cysteine and histidine residues into a cross-braced configuration [45]. Although U-box E3 ligases are also classified as RING E3 ligases, the U-box E3 ligases do not have zinc ions [46]. RING and U-box domains interact with the ubiquitin-charged E2 conjugating enzyme in a closed and active conformation and optimize the geometry of the E2-ubiquitin thioester bond and its surrounding residues to favor the nucleophilic attack by the lysine residue on the substrate, which facilitates the transfer of ubiquitin with minimal binding of the E3 ligases to ubiquitin itself [5,33,47,48,49]. RING E3 ligases are a highly diverse group functioning as monomers, homodimers, or heterodimers. Casitas B-lineage Lymphoma (CBL), C-C chemokine receptor type 4 (CCR4)-NOT transcription complex, subunit 4 (CNOT4), and RING finger protein (RNF38) are active as monomers [50,51,52], whereas cellular inhibitor of apoptosis protein 2 (cIAP2), TNF receptor associated factor 6 (TRAF6), and RNF4 are only active as homodimers [53,54,55]. Additionally, breast cancer gene 1 (BRCA1)-associated RING domain protein 1 (BARD1), B cell-specific Moloney murine leukemia virus integration site 1 (BMI1), and mouse double minute X (MDMX) become functional upon heterodimerization with a RING domain-containing partner, BRCA1, RNF2 and MDM2, respectively [56,57,58,59,60]. Further, the tripartite motif (TRIM) family of RING E3 ligases assembles homodimers, heterodimers, and oligomers via the RING domains and coiled coil regions [61]. Some RING E3 ligases, such as cullin-RING ligases (CRLs) and anaphase-promoting complex/cyclosome (APC/C), are composed of multiple subunits. CRLs, a highly diverse class of E3 ubiquitin ligases, are composed of a RING E3 (RING-box protein 1 (RBX1) or RBX2), a cullin protein (CUL1, CUL2, CUL3, CUL4A/4B, CUL5, or CUL7) as a scaffold, and a substrate receptor [62,63]. APC/C consists of a 14-subunit core complex, including a RING E3 APC11, a cullin-like subunit APC2, and a co-activator protein, cell division cycle protein 20 (CDC20) or Cdh1, for recruitment of a substrate protein [64].

With approximately 30 members in the human genome, homologous to the E6-associated protein (E6AP) carboxyl terminus (HECT)-type E3 ligases contain not only a C-terminal HECT domain that forms a thioester intermediate with ubiquitin but also various N-terminal domains that govern substrate specificity and cellular localization, both of which adopt a characteristic bilobal structure with a short hinge that enables the lobes to rotate and supports ubiquitin transfer [65]. On the basis of their N-terminal protein–protein interaction domains, HECT E3 ligases are subdivided into three groups: the neural precursor cell expressed developmentally down-regulated protein 4 (NEDD4) family, characterized by the presence of WW domains, crucial for the interaction with PY motifs in substrates; the HERC family, characterized by the presence of a HECT domain and one or more regulator of chromosome condensation 1 (RCC1)-like domains; and HECTs with other protein–protein interaction domains [66]. HECT-type E3 ligases mediate ubiquitin transfer to the substrate proteins via a two-step reaction, in which ubiquitin is transferred to an active-site cysteine on the E3 ligase to form a thioester bond with ubiquitin and then is conjugated to the substrate protein.

RING-in-between-RINGs (IBR)-RING (RBR)-type E3 ligases, as a unique family of RING–HECT hybrid E3 ligases, are characterized by a homologous sequence, consisting of two predicted RING fingers, RING1 and RING2, and a central IBR zinc-binding domain [67]. Additionally, RBR E3 ligases also possess unique N- and C-terminal flanking domains, which regulate the catalytic activities of the E3 ligases [68,69,70,71]. The human genome encodes approximately 14 RBR-type E3 ligases, including HOIL-1L interacting protein (HOIP), PRRKIN (the product of the *PARK2* gene mutated in Parkinson’s disease), the central E3 subunit of the linear ubiquitin chain assembly complex (LUBAC), two RING fingers and DRIL 1 (TRAID1), and human homologue of Ariadne (HHARI), all of which are highly conserved from yeast to humans [72,73,74,75,76]. RBR E3 ligases use an E2-binding RING domain and a second RING2 domain that contains an active cysteine residue for the formation of an E3~ubiquitin intermediate, from which ubiquitin is eventually transferred to the substrate proteins [77].

In spite of a plethora of structurally unrelated proteins, their ubiquitination is highly selective. Substrate specificity and type of polyubiquitin chains are conferred by E2 conjugating enzymes in the process of RING- and RBR-type E3 ligases-mediated ubiquitination, whereas they are governed by E3 ligases in the process of HECT-type E3 ligases-mediated ubiquitination, suggesting the importance of E2 conjugating enzymes, E3 ligases, and combinations of these two enzymes [30,66,78].

### 2.3. E4 Ubiuqitin Ligases and Ubiquitin Chain Elongation

E4 ubiquitin ligases play roles in highly processive chain elongation but not in the initial steps of ubiquitination [79,80,81,82,83]. E4 ligases not only play complementary roles to E3 ligases but also facilitate the function of E3 ligases under certain circumstances, which extends the length of polyubiquitin chains [80,84]. Ubiquitination factor E4B (UBE4B) and its isoform UBE4A are a U-box-containing RING family of ubiquitin ligases [85]. UBE4B and UBE4A are involved in the degradation of substrates through the ubiquitin fusion degradation pathway (UFD) in a similar manner to the yeast orthologue UFD2 [79,86,87]. UBE4B possesses a conserved U-box catalytic domain of about 70 amino acids, which mediates the association of UBE4B with the ubiquitin-charged E2 enzyme to facilitate attachment of a polyubiquitin chain on a selected target of UBE4B, based on its E3 and E4 ligase activity [88,89,90,91]. Additionally, UBE2A interacts with ubiquitin moieties of preformed conjugates and facilitates the elongation of the polyubiquitin chain [79,86,87].

p300 and CREB-binding protein (CBP) possess E4 ligase activity in their N-terminal regions [92,93,94]. The N-termini of p300 and CBP are cysteine-histidine-rich regions, whereas no signatures for RING, HECT, U-box, or plant homeodomain (PHD) domains can be found.

The C-terminus of the Hsc70-interacting protein (CHIP) possesses not only one U-box domain but also three tandem tetratricopeptide repeat (TPR) motifs that bind to Hsc70 and Hsc90 [95]. Similarly to yeast UFD2, CHIP acts as an E4 ligase for ubiquitin chain elongation in cooperative manner with an additional E3 ligase. CHIP is involved in ubiquitination of the PARKIN-associated endothelin receptor-like (Pael) receptor in collaboration with PARKIN [96]. Even though PARKIN can ubiquitinate the Pael receptor in vitro, only a combination of PARKIN and CHIP can sufficiently accomplish its polyubiquitination.

Yin-Yang 1 (YY1) is an E4 ligase and facilitates p53 polyubiquitination by enhancing the interaction between p53 and MDM2 [97]. Further, YY1 interacts with p300, thereby facilitating p300-mediated polyubiquitination of p53 [98].

### 2.4. Ubiquitin Code and Its Biological Significance

Ubiquitination determines the fate of substrate proteins. Functions of ubiquitination are dictated by their distinct structural topologies that are recognized by specific ubiquitin adaptors. Monoubiquitination, modification by a single ubiquitin moiety, is the most abundant modification and controls protein recognition, complex formation, allosteric regulation, endocytosis and even proteasomal degradation [99,100]. Further, ubiquitin possesses eight ubiquitination sites, seven internal lysine (K) residues (including K6, K11, K27, K29, K33, K48, and K63), and a primary amine at the N-terminal methionine (M1), all of which depend on the ubiquitination site and the length of the chains and participate in distinct homotypic polyubiquitin chain formation and chain topologies. The architecture of polyubiquitin chains is determined by the lysine acceptor site to be conjugated, the number of modified sites and the length of the added ubiquitin molecules, and combinations of these parameters have been referred to as ubiquitin code [2]. Recent discoveries have revealed that heterotypic polyubiquitin chains can be generated by the formation of mixed ubiquitin chains or branched ubiquitin chains in which ubiquitin can be modified on multiple sites [44,101,102,103,104,105]. Intriguingly, the dynamic and complex ubiquitin architecture, which includes monoubiquitination, multiple monoubiquitination, and eight different modes of homotypic and numerous types of heterotypic and branched polyubiquitin linkages, generates sophisticated and versatile ubiquitin code, thereby governing the fate of substrate proteins and providing additional regulatory nodes for cellular homeostasis [2,9,44,106].

Although the functional significance of K48- and K63-linked polyubiquitination is largely known, the biological significance of K6-, K11-, K27-, K29-, K33-, and M1-linked polyubiquitination is still far from being fully understood. Four ubiquitin molecules linked via K48 are enough to target substrate proteins to proteasomal degradation and K63-linked polyubiquitin chains serve as signals for modulation of protein trafficking, DNA repair and signal transduction [107,108,109,110,111]. Moreover, protein aggregates or dysfunctional organelles are eliminated via the autophagy–lysosome pathway, which is mediated by K6- or K63-linked chains. Additionally, K11-linked polyubiquitin chains serve as a signal for proteasome to recognize substrate, facilitating ERAD [104,112]. However, K27-, K29-, and K33-linked polyubiquitinations have begun to emerge and little is known about the biochemical information [113,114].

Interestingly, heterotypic conjugates potentially lead to combinatorial control of signaling. K11/K48-branched chains function as proteasomal priority signals and target aggregation-prone and cytotoxic proteins to proteasomal degradation [112,115]. Additionally, capping of K63-linked conjugates with M1 linkages results in the formation of M1/K63 heterotypic ubiquitin chains, which facilitates NF-κB activation [116,117,118].

## 3. “Protein Quality Control” and “Protein Quantity Control” at the ER: ERAD

**Figure 2:**
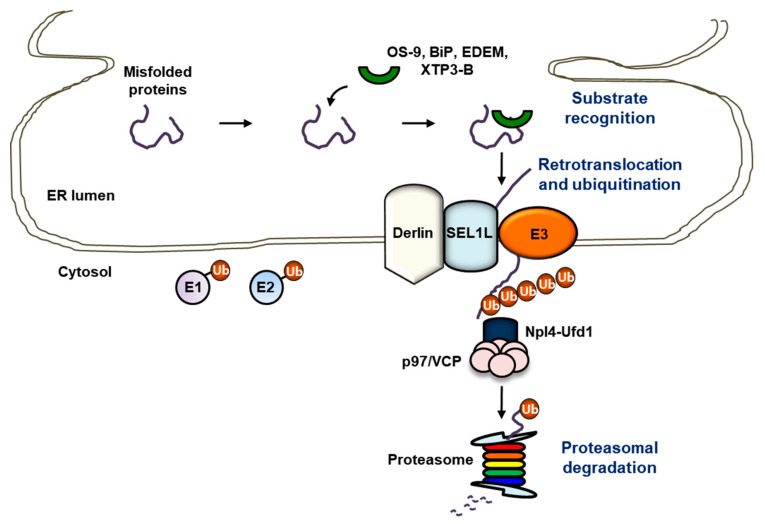
ER-associated degradation (ERAD). suppressor/enhancer of Lin12-like (SEL1L)/hydroxymethylglutaryl reductase degradation protein 1 (HRD1)-mediated ERAD is the most conserved ERAD complex in mammals. Substrate recognition: misfolded proteins are recognized by substrate recognition factors, including osteosarcoma 9 (OS-9), binding immunoglobulin protein (BiP), ER degradation-enhancing α-mannosidase like protein (EDEM), and XTP3-transactivated gene B precursor (XTP3-B). Retrotranslocation and ubiquitination: once the misfolded protein is recognized, the misfolded protein is subject to retrotranslocation and ubiquitination. Following retrotranslocation and ubiquitination, the misfolded substrate is eventually guided to the proteasome with the aid of p97/valosin-containing protein (VCP), thereby leading to its proteasomal degradation.

Despite an elaborate network within the ER for co- and post-translational folding and maturation of polypeptides, the process of protein folding and maturation is inherently error-prone and therefore misfolded proteins must be eliminated [119,120,121,122,123]. ERAD was discovered nearly 30 years ago. ERAD is highly conserved protein quality control machinery of the ER used to eliminate misfolded or unassembled proteins via the cytosolic ubiquitin-proteasome system (UPS) (Figure 2) [8,122,124,125,126]. Additionally, growing evidence indicates that ERAD also targets and fine-tunes correctly folded proteins, involving metabolically controlled enzymes, plasma membrane transporters and transcription factors, suggesting the role of ERAD in protein quantity control [127]. ERAD is a sophisticated and multistep process composed of substrate recognition, energy-dependent retrotranslocation from the ER, ubiquitination, and proteasomal degradation of substrates [128,129,130,131]. Intriguingly, deletion of key components of ERAD results in embryonic lethality in mice, pinpointing the physiological significance of ERAD in development [132,133]. Accordingly, any failure to interrupt the degradation of a specific protein in a timely manner could result in malfunction of essential cellular processes, which leads to the development of various diseases, including cancer, neurodegeneration, and metabolic diseases.

### 3.1. Substrate Recognition

Substrate recognition, the commitment step for ERAD, is accomplished by molecular chaperones and chaperone-like lectins [134]. During folding of glycoprotein, high-mannose “core” glycan possessing Glc_3_Man_9_GlcNAc_2_ (Glc: glucose, Man: mannose, GlcNAc: N-acetylglucosamine) is first appended to consensus asparagine residues within canonical N-glycosylation sites (NxS/T), which plays a leading part in monitoring conformational maturation, directing correctly folded proteins to the ER exit and directing misfolded proteins to ERAD [135]. The glycan is subsequently trimmed by lectin-type chaperone, calnexin, or calreticulin. Calnexin or calreticulin interacts with Glc_1_Man_9_GlcNAc_2_ generated by cleavage of three terminal glucose residues from the core glycans, thereby monitoring the folding of glycoproteins. To specifically generate a glycan signal for ERAD, terminally misfolded proteins must escape from the calnexin or calreticulin, which is conducted by mannosidases—including ER mannosidase I (ERManI) [136,137], ER degradation-enhancing α-mannosidase like protein 1 (EDEM1), EDEM2, EDEM3 [138,139,140,141], or Golgi-resident mannosidase α class 1C member 1 (Man1C1) [142]—that progressively eliminate terminal mannose residues from core glycans and generate an exposed α-1,6-mannose, thereby leading to association with mannose-specific lectins for ERAD [143,144]. These misfolded glycoproteins are then captured, recognized by osteosarcoma 9 (OS-9) and XTP3-transactivated gene B precursor (XTP3-B), and subsequently recruited to the protein penetration channel, retrotranslocon, for ERAD [145,146,147]. Interestingly, non-glycosylated proteins can be degraded via ERAD. Non-glycosylated proteins bind to non-lectin chaperone binding immunoglobulin protein (BiP) and are subsequently targeted to ERAD [148,149]. Additionally, EDEM1 or protein disulfide isomerase (PDI) is involved in the degradation of non-glycosylated proteins via ERAD [150,151].

### 3.2. Retrotranslocation

Energy-dependent retrotranslocation of substrates from the ER back into the cytoplasm is essential for ERAD, since the ER does not possess the components of UPS such as the E1 activating enzyme, E2 conjugating enzyme, and the proteasome [152]. Importantly, the processes of retrotranslocation, ubiquitination, and proteasomal degradation of substrates during ERAD should be tightly coupled, thereby preventing protein aggregation in an aqueous environment. p97/valosin-containing protein (VCP) is a member of the type II AAA+ protein family of ATPases and is composed of two AAA domains, D1 and D2, which are assembled in a head-to-tail manner, an N-terminal domain that functions in substrate recognition, and a C-terminal domain that associates with a large variety of adaptors [153,154,155,156]. p97/VCP plays a key role in the retrotranslocation process of nearly all ERAD substrates via coupling of ATP hydrolysis to the unfolding of ERAD substrates with the assistance of cofactors recruited through p97/VCP-binding domains, including SHP (BS1, binding segment 1) box, p97/VCP-binding region (VBR), and p97/VCP-interacting motif (VIM) [154]. Most of the p97/VCP cofactors also contain ubiquitin-binding domains (UBDs), thereby leading to the direct association with ubiquitinated substrates. Interestingly, p97/VCP in cooperation with nuclear protein localization protein 4 (Npl4) and ubiquitin fusion degradation protein 1 (Ufd1) can generate a driving force for the retrotranslocation of substrates for ERAD [157]. To summarize, the ERAD substrate is slightly exposed to the ER surface through the retrotranslocon, subject to E3 ubiquitin ligase-mediated polyubiquitination and subsequently further retrotranslocated by the p97/Npl4/Ufd1 complex, highlighting that the two processes of substrate retrotranslocation and polyubiquitination by ERAD E3 ubiquitin ligases should be tightly coupled and that p97/VCP functions as a scaffold to recruit the various factors involved in ERAD and to regulate ubiquitination at sites of retrotranslocation [129,158,159,160,161,162].

### 3.3. Ubiquitination and E3 Ubiquitin Ligases

Protein quality control E3 ubiquitin ligases involved in ERAD determine the specificity, plasticity, and efficiency of ERAD and subsequently fine-tune proteostasis. As unrestrained degradation would destroy cellular homeostasis, it is crucial that E3 ligases ensure only the specificity required to eliminate misfolded proteins, while leaving their correctly folded counteracts intact. Moreover, E3 ligases usually need to target similarly misfolded and potentially cytotoxic factors, suggesting the necessity of a delicate balance between specificity and plasticity. Approximately 40 ERAD E3 ubiquitin ligases have been identified in mammals and three in yeast to date [163]. However, most of the ERAD E3 ubiquitin ligases are poorly characterized and only a few substrates have been identified (Table 1).

Several ERAD E3 ubiquitin ligases, including glycoprotein 78 (gp78), also known as autocrine motility factor receptor (AMFR)), hydroxymethylglutaryl reductase degradation protein 1 (HRD1), membrane-associated RING C3HC4 finger 6 (MARCHF6), and RING finger protein 5 (RNF5), are transmembrane proteins [129,164,165,166,167,168,169,170], while another set of E3 ubiquitin ligases, including CHIP, SMAD ubiquitination regulatory factor 1 (Smurf1), neuregulin receptor degradation pathway protein 1 (Nrdp1)/fetal lever ring finger (FLRF), PARKIN, and the Skp, Cullin and F-box containing complex (SCF) with the F-box proteins Fbx2, Fbx6, or beta-transducin repeat-containing proteins 1 and 2 (β-TrCP1/2), are localized in the cytoplasm and involved in ERAD [96,171,172,173,174,175,176]. ERAD E3 ubiquitin ligases may accomplish polyubiquitination of ERAD substrates not only in cooperation with other E3 ubiquitin ligase-mediated multiple monoubiquitinations or E4 ubiquitin ligase-mediated extensions after initial monoubiquitination but also by the aid of sequential rounds of ubiquitination and deubiquitination, raising the possibility that various strategies have evolved for the optimal efficiency of ERAD [15,177,178,179,180].

Interestingly, for the degradation of ERAD substrates, mixed K48/K11-linked polyubiquitin chains are appended, facilitating the association with downstream components of ERAD machinery [181]. Further, DUBs fine-tune the timing of ERAD by trimming polyubiquitin chains on ERAD substrates [182]. Notably, ubiquitin can be targeted to thiol or hydroxyl side chains in cysteine, serine, and threonine residues on the substrate proteins, suggesting that the diversity in amino acids modified by ERAD-specific E2 conjugating enzyme/E3 ligase pairs provides the flexibility to ubiquitinate ERAD substrates [37,183].

#### 3.3.1. HRD1

HRD1-mediated ERAD is the most conserved and best characterized branch of mammalian ERAD and it is indispensable for versatile homeostatic processes, including energy metabolism, food intake, systemic water balance, and immune cell development [184,185,186,187,188,189,190,191,192]. HRD1 is involved in the ubiquitination of not only glycosylated but also non-glycosylated ERAD substrates. HRD1 commonly targets substrates for degradation in a suppressor/enhancer of Lin12-like (SEL1L)-dependent manner. SEL1L is crucial not only for the transfer of substrates to HRD1 but also for the stabilization of HRD1, indicating the key role of SEL1L in the recruitment, retrotranslocation, and ubiquitination of ERAD substrates [146,193,194,195,196]. SEL1L also recruits recognition factors, including EDEMs, ERdj5, OS-9, PDI, and XTP3-B, to the retrotranslocon for ERAD substrates [197]. Further, SEL1L functions as a scaffold to generate a complex with ancient ubiquitous protein 1 (AUP1), degradation in endoplasmic reticulum protein (Derlin)-1, Derlin-2, ubiquitin regulatory X (UBX) domain containing protein 8 (UBXD8), and VCP-interacting membrane protein (VIMP) [198,199,200,201,202], which recruits the p97/VCP and leads to substrate retrotranslocation. Derlin-1, Derlin-2, or Derlin-3 interact with ERAD substrates and target ERAD substrates to HRD1 and p97/VCP, indicating that Derlins work together as a part of the retrotranslocon channel [203,204,205], although the role of Derlins needs further characterization in mammalian systems. Interestingly, auto-ubiquitination of HRD1 facilitates retrotranslocation of misfolded protein substrates, suggesting that HRD1 might be a protein-conducting channel opened by ubiquitin moiety [206].

B cell development-specific pre-B cell receptor (pre-BCR), B-lymphocyte-induced maturation protein 1 (BLIMP1), Fas, inositol-requiring enzyme 1 (IRE1), NF-E2-related factor 2 (NRF2), p53, peroxisome proliferator activated receptor γ coactivator-1 β (PGC1β), and pro-opiomelanocortin (POMC) have been identified as endogenous substrates for SEL1L/HRD1-mediated ERAD [186,188,189,196,207,208,209]. Notably, HRD1-mediated ERAD functions to ensure optimal levels of substrate proteins not only qualitatively but also quantitatively. The HRD1-mediated degradation of IRE1 constitutively fine-tunes IRE1 activity under normal physiological conditions, thereby limiting the amplitude and duration of IRE1 activity and maintaining cellular homeostasis [189]. Interestingly, deletion of either SEL1L or HRD1 in various tissues and cell types leads to a profound increase in the level of IRE1 protein, whereas its mRNA levels are unchanged [189]. However, it remains elusive as to whether IRE1 is subjected to ERAD-mediated quality control to eliminate misfolded IRE1 or to ERAD-mediated quantity control to ensure adequate the protein level of IRE1. Additionally, HRD1-mediated constitutive degradation of pre-BCR in developing B cells regulates the abundance of surface pre-BCR, thereby restraining pre-BCR-mediated signaling during the transition from the large to small pre-B cell stage [188,210]. Moreover, HRD1-mediated constitutive degradation of misfolded pro-arginine vasopressin (AVP) maintains systemic water balance [186]. Mice depleted of SEL1L in AVP neurons develop polyuria and polydipsia, suggesting the pathophysiological importance of the constitutive ERAD in modulating the maturation and abundance of specific ER proteins and fine-tuning their activities. Recently, SEL1L/HRD1-mediated ERAD has been reported to target transforming growth factor beta (TGF-β) receptor I for proteasomal degradation, resulting in the control of β-cell identity via TGF-β signaling [211].

A variety of studies have suggested HRD1 as a critical immune regulator. HRD1-mediated ERAD eliminates major histocompatibility complex (MHC) class I heavy chains that fail to acquire their native conformation in complex with β2-microglobulin and peptide, suggesting the role of HRD1 as a positive regulator for the peptide antigen presentation by MHC I molecules [212,213,214]. Additionally, HRD1 directly recognizes, ubiquitinates, and mediates proteasomal degradation of BLIMP1, a transcription co-repressor that inhibits the transcriptional activity MHC II transcription activator (CIITA) [215,216]. Further, HRD1 attaches ubiquitin to the serine side chains on T-cell receptor α (TCRα) [183] and to serine, threonine, and lysine residues on immunoglobulin nonsecreted Ig light chain (NS1LC) [37], suggesting the importance of HRD1 for the flexibility of ubiquitination during ERAD to cope with cellular stresses.

HRD1 is involved in anti-apoptosis [217]. HRD1-mediated degradation of IRE1 via ERAD prevents ER stress-induced cell death [189,218]. Additionally, HRD1 facilitates cell cycle progression and anti-apoptosis by mediating p53 ubiquitination [208]. HRD1 has also been reported to mediate ubiquitination and subsequent degradation of Fas, thereby resulting in the prevention of B-cell apoptosis [209].

Although the underlying mechanisms by which HRD1 recognizes nuclear substrates remain elusive, HRD1 may directly mediate the degradation of nuclear transcription factors, including BLIMP1, NRF2, and PGC1β [192,207,215,219], revealing intriguing regulatory cascades from protein degradation at the ER membrane to gene transcription in the nucleus.

#### 3.3.2. RNF45/gp78/AMFR

Gp78 is another major ERAD E3 ubiquitin ligase in mammals [166]. Five transmembrane domains reside at the N-terminus of gp78 and the RING finger domain is localized at the C-terminal cytoplasmic tail [220,221]. Additionally, the E2 ubiquitin-conjugating enzyme, UBE2G2 binding region (G2BR) of gp78 associates with UBE2G2, which leads to conformational alterations in UBE2G2 and an increase in the affinity of UBE2G2 for gp78 [222,223]. In a similar manner to HRD1, gp78 recruits UBXD2 or UBXD8, thereby functioning as a bridge to connect p97/VCP to the ER membrane. Alternatively, gp78 directly interacts with p97/VCP via its VIM domain, which is localized at the C-terminus [160]. Interestingly, USP13 associates with gp78 to attenuate polyubiquitination of ubiquitin-like protein 4A (UBL4A), a part of the Bag6 complex that helps chaperone retrotranslocated ERAD substrates to the proteasome, thereby protecting the Bag6 complex from proteasomal degradation and maintaining an adequate ERAD pathway, highlighting the importance of the associated DUB activity during ERAD [224].

Gp78 plays an essential role in fine-tuning the levels of proteins quantitatively in response to specific signals. Sterol accumulation alters transmembrane regions of 3-hydroxy-3-methyl-glutaryl-coenzyme A (CoA) reductase (HMGCR), which is an ER membrane-anchored protein, and catalyzes the rate-limiting step of sterol biosynthesis, in which HMG-CoA is reduced to mevalonate [225,226] and facilitates the binding of HMGCR to ER membrane-embedded insulin-induced genes 1/2 (INSIG-1/2) proteins, thereby resulting in the association of HMGCR with gp78 and subsequent ubiquitination and proteasomal degradation of HMGCR [221]. Interestingly, viral or microbial DNA promotes the association of gp78 with INSIG-1 and stimulator of interferon genes (STING) and facilitates K27-linked polyubiquitination of STING, which results in the association of STING with TANK-binding kinase 1 (TBK1) and the translocation of STING to perinuclear microsome, thereby leading to innate immune response [227].

It has been suggested that Gp78 has neurobiological functions. Gp78 mediates ubiquitination and subsequent degradation of disease-associated aggregative proteins, such as superoxide dismutase-1 (SOD1), ataxin-3, and the mutant form of huntingtin [228,229]. Further, gp78 specifically associates with the C-terminal region of unglycosylated prion proteins that cause bovine spongiform encephalopathy and Creutzfeltd–Jacob diseases and facilitates ubiquitination and subsequent proteasomal degradation of prion proteins [230].

Interestingly, gp78 also functions as an E4 ubiquitin ligase in a cooperative manner with other ERAD E3 ubiquitin ligases [177]. For example, gp78 serves as an E4 ubiquitin ligase for RNF5-mediated ubiquitination of cystic fibrosis transmembrane conductance regulator (CFTR)∆F508 in ERAD [168,177].

#### 3.3.3. MARCHF6/TEB4/RNF176

MARCHF6, akin to Doa10 in yeast, is a large multi-pass E3 ubiquitin ligase embedded in the membranes of the ER and plays a central role in sterol and lipid metabolism [167,231,232,233]. MARCHF6 is characterized by two functional domains: the catalytic N-terminal RING domain and the C-terminal element (CTE) that is involved in its self-regulation [167,234]. MARCHF6 cooperates with the E2 ubiquitin-conjugating enzyme, UBE2G1, and mediates ubiquitination and proteasomal degradation of ERAD substrates with the aid of HSC70, DNAJB12, HDJ2, and UBXD8 [235,236,237,238,239].

HMGCR and squalene epoxidase (SQLE, also known as squalene monooxygenase) are controlled by MARCHF6-mediated ERAD, although SQLE is widely considered as the canonical substrate for MARCHF6 [240,241]. SQLE is subjected to ERAD when additional cholesterol synthesis is unnecessary. MARCHF6 and the E2 conjugating enzyme Ube2J2 facilitate degradation of SQLE via ERAD [242]. Sterol-dependent ERAD is mediated by an N-terminal SQLE degron containing an amphipathic helix, which allows sterol-dependent recognition of SQLE by MARCHF6 [242,243,244]. Additionally, Perilipin-2 (PLIN2), mutant versions of Niemann–Pick disease type C intracellular cholesterol transporter 1 (NPC1), and bile salt export pump (BSEP) are identified as target substrates for MARCHF6 [245,246,247]. Interestingly, MARCHF6 preferentially targets N-terminally acetylated residues in PLIN2, suggesting that MARCHF6 could degrade substrates via the Ac/N-end rule pathway, in which N-terminally acetylated residues lead to destabilization of substrates [245,248]. Further, MARCHF6 indirectly affects sterol regulatory element binding proteins (SREBP) and liver X receptor (LXR), both of which are master transcription factors for the control of cholesterol levels [249].

MARCHF6 itself has a short half-life due to its autoubiquitination. Interestingly, USP19, an ER-resident DUB, and cholesterol enhance the stability of MARCHF6 [250,251].

#### 3.3.4. RNF5/RMA1

RNF5 is a membrane-anchored E3 ubiquitin ligase localized not only in the ER but also in mitochondrial membranes [168,169,170]. RNF5 functions sequentially with CHIP to degrade misfolded CFTR [168]. RNF5 recognizes folding defects in CFTR∆F508 coincident with translation, while CHIP acts on CFTR∆F508 post-translationally. Further, RNF5-mediated ubiquitination-dependent relocalization of the proteasome adaptor protein JNK-associated membrane protein (JAMP) contributes to the recruitment of the proteasomes in the vicinity of the ER [252].

### 3.4. Delivery and Degradation

p97/VCP functions as a bridge to connect retrotranslocated substrates to cytoplasmic cofactors for further processing and subsequent degradation of ERAD substrates, suggesting that p97/VCP is closely associated with the proteasome-mediated degradation of substrates [154]. Cytoplasmic N-glycanase 1 (NGly1), a deglycosylating enzyme, is recruited to retrotranslocon complexes via direct association with p97/VCP and cleaves N-linked glycans from retrotranslcated ERAD substrates [253,254]. DUBs, including Ataxin-3, ovarian tumor family of deubiquitinating enzyme 2 (OTUD2, also known as YOD1), USP13, and valosin-containing protein p97/p47 complex-interacting protein p135 (VCIP135) interact with p97/VCP either in a direct or indirect manner and subsequently deubiquitinate ERAD substrates [255,256,257]. OTUD2 in cooperation with p97/VCP may facilitate the efficient retrotranslocation of ubiquitinated ERAD substrates. Notably, the impairment of p97/VCP-associated deubiquitination or expression of dominant-negative OTUD2 impedes retrotranslocation and subsequent degradation of ERAD substrates [255], indicating that the sequential rounds of ubiquitination and deubiquitination are indispensable for an efficient ERAD process. USP13 interacts with p97/VCP, Ufd1, Npl4, and UBXD8 [256]. Interestingly, the depletion of USP13 leads to the accumulation of ERAD substrates. The role of ataxin-3 in ERAD remains to be elucidated. Ataxin-3 has been suggested to act after retrotranslocation of ERAD substrates [257]. Alternatively, ataxin-3 may cleave off ubiquitin from polyubiquitinated ERAD substrates, which increases the half-life of protein and provides time for folding of ERAD substrates [258].

Retrotranslocated ERAD substrates should be rapidly eliminated to prevent the formation of protein aggregates in the cytoplasm. Bag6, ubiquitin-like protein 4A (Ubl4A), and the transmembrane domain recognition complex 35 (Trc35) co-chaperone small glutamine rich TPR-containing protein α (SGTA) form a chaperone complex, which prevents the formation of protein aggregates [259]. Bag6 oligomerizes through its proline-rich domain. Interestingly, the proline-rich domain is sufficient for the association of Bag6 with the hydrophobic segments of misfolded proteins, thereby leading to the maintenance of misfolded proteins in a soluble state [260]. Moreover, the holdase activity of Bag6 is indispensable for maintaining retrotranslocated substrates in a competent state for proteasome-mediated degradation [261]. Interestingly, Bag6 also interacts with the proteasome and adaptor proteins of the proteasome, resulting in the transfer of retrotranslocated substrates to proteasome for ERAD.

## 4. “Protein Quality Control” at the ER: ER-Phagy

While most of the misfolded proteins in the ER are efficiently eliminated via ERAD, the pore size of the retrotranslocon may preclude misfolded oligomeric or aggregated proteins from being exported from the ER, which leads to the development of another mechanism, selective autophagic degradation of the ER, referred to as ER-phagy, which eliminates these toxic substrates, thereby maintaining proteostasis. The term ER-phagy was first used upon the discovery of ER whorls, which occur after unfolded protein response (UPR)-associated lipid synthesis in yeast [262,263]. ER-phagy is then initiated to utilize the core autophagy machinery and eliminate ERAD-resistant misfolded proteins [17,264].

**Figure 3:**
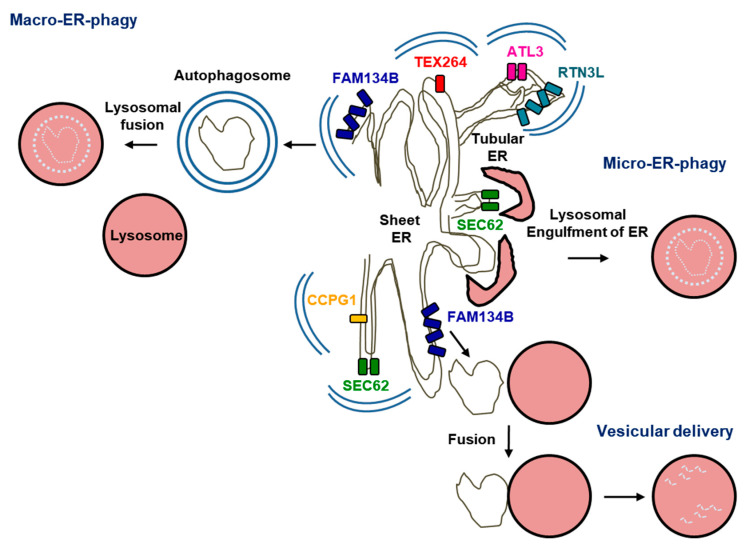
Different types of ER-phagy. In macro-ER-phagy, fragments of the ER together with cytoplasmic components are enclosed by autophagosome, which fuses with lysosomes and is subsequently degraded. In micro-ER-phagy, a small portion of the ER is invaginated by lysosomes and subsequently degraded. In vesicular delivery, ER-derived vesicles containing misfolded proteins bud off from the ER and fuse directly with lysosomes.

The identification of versatile ER-phagy receptors responsible for substrate recognition in mammals, including family with sequence similarity 134 member B (FAM134B), reticulon domain containing family member 3 long (RTN3L), secretory 62 homologue (SEC62), cell-cycle progression gene 1 (CCPG1), atlastin 3 (ATL3), and testis-expressed protein 264 (TEX264), has advanced our understanding of ER-phagy [265,266,267,268,269,270,271]. By definition, the ER-phagy receptors can directly mediate the recognition of the ER membrane by phagophores or lysosomes. The ER-phagy receptors are either directly ER membrane-anchored, through insertion of a part of the polypeptide into the ER membrane from the cytosolic side, or bona fide transmembrane proteins [272,273]. ATL3, FAB134B, and RTN3L possess an intramembrane region (IM) lacking an ER luminal domain. In contrast, CCPG1, SEC62, and TEX264 are transmembrane proteins with a cytosolic, ER membrane, and ER luminal domains. The ER tightly binds to the outer and inner membranes of autophagosomes to form ER–autophagosome contacts, through which the ER can supply lipids and other components for autophagosome expansion [274,275,276]. ER-phagy can be classified into three distinct pathways on the basis of topology (Figure 3) [277]. During macro-ER-phagy, autophagosomes enclose fragments of the ER and fuse with lysosomes to eliminate the internal material harboring the ER [278,279]. During micro-ER-phagy, lysosomal membranes invaginate and pinch off parts of the ER into the lysosomal lumen [272,280]. Lastly, during vesicular delivery, lysosomes can directly fuse with ER-derived vesicles for degradation [17,281].

### 4.1. Macro-ER-Phagy

#### 4.1.1. A Conceptual Overview of Macro-ER-Phagy

Since the discovery of sequestration of the ER by autophagosomes over 50 years ago [282], it has remained uncertain whether this process involves selective or random sequestration of the ER. However, the identification of ER-phagy receptors indicates that ER-phagy involves the selective recognition of the ER to ensure selective autophagy. Importantly, ER-phagy is a type of selective autophagy for the removal of misfolded protein aggregates to maintain proteostasis. To date, six ER-phagy receptors, FAM134B, RTL3L, CCPG1, SEC62, TEX264, and ATL3, have been identified in mammals [265,266,267,268,269,270]. These ER-phagy receptors are ER membrane-anchored proteins and possess at least one microtubule-associated protein 1A/1B-light chain 3 (LC3)-interacting motif (LIR), which leads to the association with autophagosomal LC3 proteins. Additionally, all ER-phagy receptors except for ATL3 possess long intrinsically disordered regions (IDRs) between the LIR and the ER transmembrane domain. IDR functions as a bridge connecting the ER and autophagosomal membranes [277].

Several functional characteristics of the ER-phagy receptors have been reported [277]. The ER-phagy receptors can mediate the degradation of different subdomains of the ER, including nuclear membranes, the sheet ER, the tubular ER, and the ER exit site (ERES). Additionally, individual ER-phagy receptors can facilitate specific stimuli-triggered ER-phagy. ER-phagy is induced upon ER stress or starvation conditions. Further, some ER-phagy receptors contain ER-fragmentation activity, which cleaves a portion of the ER network before or during sequestration into autophagosomes. Reticulon homology domains (RHDs) consist of two short hairpin transmembrane domains, which can generate membrane curvature by partially inserting into the membrane, thereby leading to an increase in the surface area of the cytosolic leaflet [283]. Moreover, association of ER-phagy receptors with membrane-tethered LC3 is indispensable not only for linkage of the ER to the phagophore but also for concomitant membrane reshaping to pack into autophagosomes [265]. Lastly, ER-phagy receptors are distributed differently in a tissue-specific manner. FAM134B, TEX264, SEC62, and ATL3 show ubiquitous expression, whereas CCPG1 shows predominant expression in the pancreas, kidney, and liver [268,271,284].

#### 4.1.2. Macro-ER-Phagy and Protein Quality Control

FAM134B, also known as reticulophagy regulator 1 (RETREG1), is classified as an IM ER-resident protein characterized by the presence of LIR and RHD [265]. RHD mediates post-translational insertion of FAB134B into the lipid bilayer. N- and C-termini of FAM134B are cytosolic and LIR is at the C-terminus. FAM134B localizes at the edge of ER sheets via RHDs and promotes membrane curvature during autophagosome formation, thereby mediating starvation-induced ER-phagy [265]. In addition to RHDs, two amphipathic helices of FAM134B are involved in membrane curvature [285,286]. The LIR motif within FAM134B recruits LC3 and gamma-aminobutyric acid receptor-associated protein (GABARAP) into the growing phagophores. Intriguingly, a group of misfolded proteins has been identified as FAM134B cargoes. FAM134B-mediated ER-phagy eliminates ERAD-resistant protein aggregates such as Niemman–Pick type C disease protein (NPC), mutant forms of type I procollagen (PC1), and the collagen chaperone HSP47 [246,281,287,288,289]. The folding process of PC is complicated and approximately 15% of newly synthesized procollagens is degraded [290]. Calnexin not only recognizes luminal misfolded PCs but also interacts with FAM13B, thereby leading to the degradation of misfolded PCs via ER-phagy [288]. Given that FAM134B does not have an ER luminal domain, calnexin is essential for the selective interaction of FAM134B with PCs. Further, the TM domain of calnexin associates with the RHD of FAB134B, indicating the role of calnexin as a bridge connecting ER luminal PCs to FAM134B [288].

RTN3L, a long splice isoform of RTN3, is an ER tubules resident protein. RTN3L possesses C-terminal RHD for anchorage of RTN3L to the ER from the cytosolic face of the membrane. RTN3L possesses an extended N-terminus containing six distinct LIR motifs spaced at uneven intervals and oligomerizes to engage and cluster LC3, thereby leading to the formation of autophagosomes at the ER tubules [267,291]. RTN3L is upregulated during starvation and facilitates starvation-induced ER-phagy. RTN3L is not involved in either LC3 lipidation or p62 degradation, thus indicating its selective role in ER degradation rather than in general autophagy. Interestingly, RTN3L is involved in the degradation of misfolded proinsulin protein aggregates via ER-phagy and the enhanced expression of RTN3L is involved in the relief of ER aggregates and in the export of wild-type proinsulin [292].

CCPG1 is a type II single-pass transmembrane protein and possesses both LIR- and focal adhesion kinase family interacting protein of 200 kDa (FIP200)-interacting regions, which associate with LC3 and FIP200, respectively [268]. In contrast to FAM134B and RTN3L, CCPG1 possesses an extensive C-terminal ER luminal region of undefined structure and an IDR at the N-terminus. Although CCPG1 does not have intrinsic ER fragmentation activity, it is likely that accumulation of IDR-containing CCPG1 induces molecular crowding at the site facing autophagosomes, resulting in the membrane curvature and scission [293,294]. Even though it is not clear whether CCPG1 selectively recognizes aggregated or misfolded proteins, upregulation of CCPG1 upon ER stress is known to be involved in the removal of the portions of the peripheral ER, resulting in the reduction of protein aggregation. Particularly, CCPG1 deficiency in the ER leads to numerous luminal protein inclusions and subsequent elevation of UPR [268].

TEX264 is a single-pass transmembrane protein and possesses a negligible N-terminal luminal region of approximately five amino acids and a C-terminal cytosolic LIR [269,271]. TEX264 is involved in approximately 50% of starvation-induced autophagic flux from the ER.

In addition to the transmembrane ER-phagy receptors, p62 could also mediate ER-phagy. In mouse liver, xenobiotics-induced excess ER is eliminated by p62-mediated autophagy [295]. IRE1 associates with p62 and cytosolic ubiquitin-binding receptors, optineurin and neighbor of BRCA1 gene 1 (NBR1), suggesting the role of ER-phagy in the sequestration of active IRE1-enriched ER subdomains to terminate UPR. Further, the association of p62 with ubiquitinated TRIM13 facilitates ER-phagy [296].

### 4.2. Micro-ER-Phagy

Micro-ER-phagy was first discovered as piecemeal micronucleophagy in yeast [297]. Additionally, ER-targeted microautophagy is also observed in yeast. Dithiothreitol-induced ER stress mediates formation of ER whorls, which are selectively delivered to the vacuole by microautophagy [263,280]. Microautophagy in yeast requires the endosomal sorting complexes required for transport (ESCRT) machinery for scission of vacuolar membrane, whereas this process does not require core autophagy factors and macro-ER-phagy receptors [298].

In mammals, micro-ER-phagy is involved during recovery from cyclopiazonic acid-induced ER stress, in which ER-derived vesicles are directly engulfed by endolysosomes [299]. Compared to micro-ER-phagy in yeast, micro-ER-phagy in mammals requires SEC62 and the LC3 lipidation system components, including autophagy-related protein 4B (ATG4B), ATG7, and AYG16L1. However, in the process of SEC62-dependent micro-ER-phagy, LC3 is present on SEC62-decorated ER rather than on lysosomes. Therefore, further studies are warranted to investigate the incorporation of selectivity during micro-ER-phagy. Additionally, mammalian micro-ER-phagy requires CHMP4B, an ESCRT-III component. Importantly, a subset of proteins can be eliminated via micro-ER-phagy. A disease-causing mutant form of PCs (G610C) accumulates at the ERES, a representative of very specialized ER zones for the transport of cargo proteins from the ER to the Golgi apparatus [300] and in which ubiquitin, p62, LC3, ATG14, ATG4, and COPII also accumulate, and leaves the ER through the ERES via COPII-coated vesicles, thereby resulting in the direct engulfment of the mutant form of PCs by lysosomes via micro-ER-phagy [301]. Notably, this micro-ER-phagy occurs at an early stage of PC trafficking, thereby ensuring that only adequately folded PCs enter the secretory pathway.

### 4.3. Vesicular Delivery

Vesicular delivery, a pathway for lysosomal degradation of ER-derived single-membrane vesicles, has been recently proposed [17,281]. Calnexin segregates α1-antitrypsin Z (ATZ) polymers into subdomains of the ER and is involved in the formation of a complex with LC3 and FAM134B, resulting in the generation of local membrane curvature and scission and subsequent formation of ER-derived vesicles. Further, ER-resident SNARE syntaxin 17 (STX17) and lysosomal SNARE vesicle-associated membrane protein 8 (VAMP8) mediate fusion of the ER-derived vesicles with endolysosomes. Interaction of FAM134B with LC3 at vesicle-lysosome contact sites drives fusion, indicating the role of LC3 in the recognition of vesicular receptor. The vesicular delivery does not require autophagy initiation complex components, including ATG13, ATG9, FIP200, and unc-51 like autophagy activating kinase (ULK1/2), but requires LC3-conjugation machinery, which could be important for the docking of ER-derived vesicles into endolysosomes [302]. However, ATZ has also been reported to be degraded by lysosomes in an ATG conjugation-dependent manner, suggesting the requirement in some ATGs for this novel pathway [303,304,305].

## 5. Brief Linking of “Protein Quality Control” at the ER to Neurodegenerations

In the last decade, multiple studies have implicated the pivotal roles of the ER in the progression of neurodegenerative diseases, including Alzheimer’s disease (AD), amyotrophic lateral sclerosis (ALS), Parkinson’s disease (PD), and Gaucher disease (GD) [306,307,308,309,310]. Although it is still controversial whether ER stress and the subsequent protein quality control at the ER are closely linked to neurodegenerative diseases, misfolded proteins and their aggregates are implicated in the pathogenesis of neurodegenerative diseases.

PD is a progressive neurodegenerative disease and is characterized by the aggregates accumulation of misfolded α-synuclein (α-SYN) fibrils into proteinaceous inclusions in Lewy bodies (LB) or Lewy neurites [311]. Toxicity resulting from the overexpression of α-SYN is associated with ER stress and activation of the UPR [312,313,314,315,316,317], which subsequently facilitates ER stress-induced dopaminergic neuronal death [312,318]. Interestingly, α-SYN aggregates associate with BiP, thereby leading to the activation of the UPR [314,319]. Additionally, α-SYN has been reported to be abundant in ER/microsome fractions of human and mice PD brain tissues, resulting in the ER stress-associated accumulation of polyubiquitin chains and the activation of caspase-12 [315]. Moreover, accumulation of aggregates of α-SYN inhibits the ER–Golgi trafficking by directly binding to ras-associated binding 1 (RAB1) GTPase, which leads to ER stress [313,320,321]. Intriguingly, accumulation of aggregates of α-SYN destroys ER Ca^2+^ homeostasis, thereby leading to the induction of ER stress. α-SYN aggregates activate sarco/endoplasmic reticulum Ca^2+^-ATPase (SERCA) in neurons, which results in alteration of ROS production and calcium metabolism and cell death [322,323].

PARKIN, an E3 ubiquitin ligase, is involved in a large variety of cellular processes related to PD [324,325]. Interestingly, ER stress-induced upregulation of PARKIN plays a neuroprotective role against ER stress [325]. Additionally, the Pael receptor, which is involved in ER stress-induced cell death, is a substrate of PARKIN [307,326]. Further, PARKIN overexpression in drosophila models results in an increase in K48-linked polyubiquitination and a decrease in protein aggregation [327].

HRD1 attenuates 6-hydroxydopamine (6OHDA)-induced cell death in dopaminergic neuroblastoma cells [328].

GD is the most common lysosomal storage disorder (LSD) and is an autosomal recessive sphingolipidosis caused by mutations in a lysosomal enzyme, glucocerebrosidase (GCase), or its activator protein, saposin C, which functions in hydrolysis of glucosylceramide (GlcCer) to ceramide and glucose [308,329]. Therefore, GD is characterized by accumulation of GlcCer. Interestingly, accumulation of GlcCer in neurons leads to an increase in ER Ca^2+^ release through ryanodine receptors (RyRs) and subsequent neuronal cell death [330,331]. Interestingly, RyR antagonist dantrolene corrected deregulated Ca^2+^ signaling and autophagy defects in a GD mouse model, suggesting the relation between Ca^2+^ and autophagy in GD [332,333].

Misfolded mutants of GCase are retained in the ER and are not efficiently transported from the ER to the lysosomes, which are subject to ERAD [334,335]. Interestingly, mutants of GCase show variable degrees of ER retention and subsequent ERAD, which is correlated with GD severity [336].

## 6. Concluding Remarks and Future Perspectives

The ER is an interconnected organelle that plays fundamental roles in the biosynthesis, folding, stabilization, maturation, and trafficking of secretory and transmembrane proteins. It is the largest organelle and critically modulates nearly all aspects of life, including food intake, water balance, growth, metabolism, and immunity. Therefore, the ER undergoes continuous reconstruction to maintain its function and integrity. Unfortunately, even with an elaborate network to ensure adequate protein folding and assembly, misfolding and subsequent formation of protein aggregates can occur quite frequently, which could be a primary pathogenic mechanism in multiple human disorders. Therefore, mammals have developed two sophisticated guardian pathways, ERAD and ER-phagy, for the maintenance of proteostasis and organismal homeostasis. However, the relative contributions of these two guardian pathways remain still elusive. Many proteins can be eliminated by both pathways, suggesting that ERAD and ER-phagy are not mutually exclusive [16,246,337]. Given that more than 8000 proteins pass through the ER in a single human cell [338], the diversity as well as the flexibility of the two guardian pathways must be getting more complex and elaborate.

The field of ubiquitin has been extensively studied in the last several decades and a large number of studies aim to improve our understanding of the complex nature of the ubiquitin network. Given that E3 ligases are dysregulated in various processes of pathogenesis, E3 ligases have emerged as promising therapeutic targets for the treatment of diseases. Compared to ERAD, the involvement of specific E3 ubiquitin ligase and ubiquitination during the process of ER-phagy remains largely unknown. p62 has been shown to engage ubiquitinated cargos with autophagosomes during ER-phagy [339]. More recently, the association of p62 with TRIM13 has been reported to mediate ER-phagy [296]. Addressing outstanding mechanistic questions regarding E3 ubiquitin ligases and the subsequent ubiquitination of ER proteins during ER-phagy is important as the recruitment of ubiquitin-binding proteins for recognition by the phagophore could expand fundamental knowledge about this fascinating process.

Deregulation of protein quality control as well as protein quantity control at the ER and the subsequent failure in the re-establishment of proteostasis are closely associated with various human diseases, including cancer, cardiovascular diseases, immune diseases and neurodegenerative diseases [119,120]. Therefore, more emphasis must be placed on interrogating how cells manage the complexity of the ubiquitin code and how the cellular ubiquitin dynamics are fine-tuned in the ER, as this could lead to the identification of specific biomarkers, such as up- or downregulated UPR markers and dysregulated E3 ubiquitin ligases, for multiple human diseases and the characterization of new targets for drug development.

## Figures and Tables

**Table 1:** List of ERAD E3 ubiquitin ligases and their identified substrates.

E3 Ubiquitin Ligases (Mammalian)	Substrates	Notes
HRD1	pre-BCR, BLIMP1, Fas, IRE1, NRF2, p53, PGC1β, AVP, TGF-β receptor I, TCR-α, MHC I	Homolog of yeast Hrd1
MARCHF6	SQLE, HMGCR, PLIN2, BSEP	Homolog of yeast Doa10
gp78	CFTR, HMGCR, SOD1, ataxin-3, huntingtin, prion	Homolog of yeast Hrd1
RNF5	CFTR, JAMP	Integral ER membrane-resident
CHIP	CFTR, Pael-receptor	Cytoplasmic
PARKIN	Pael-receptor, GCase	Cytoplasmic
Smurf1	WFS1	Cytoplasmic/nuclear
Nrdp1	ErbB3	Cytoplasmic
SCF^Fbx2^	TCR-α, CFTR	Cytoplasmic/ER membrane-associated
SCF^Fbx6^	TCR-α	Cytoplasmic/ER membrane-associated
SCF^β-TrCP^	CD4, Tetherin	Cytoplasmic

Hydroxymethylglutaryl reductase degradation protein 1 (HRD1); membrane-associated RING C3HC4 finger 6 (MARCHF6), glycoprotein 78 (gp78); RING finger protein 5 (RNF5); C-terminus of the Hsc70-interacting protein (CHIP); SMAD ubiquitination regulatory factor 1 (Smurf1); neuregulin receptor degradation pathway protein 1 (Nrdp1); Skp, Cullin and F-box containing complex (SCF); pre-B cell receptor (pre-BCR); B-lymphocyte-induced maturation protein 1 (BLIMP1); inositol-requiring enzyme 1 (IRE1); NF-E2-related factor 2 (NRF2);peroxisome proliferator activated receptor γ coactivator-1 β (PGC1β); pro-arginine vasopressin (AVP); transforming growth factor beta (TGF-β); T-cell receptor alpha (TCR-α); major histocompatibility complex I (MHC I); squalene epoxidase (SQLE); 3-hydroxy-3-methyl-glutaryl-coenzyme A (CoA) reductase (HMGCR); perilipin-2 (PLIN2); bile salt export pump (BSEP); cystic fibrosis transmembrane conductance regulator (CFTR); superoxide dismutase-1 (SOD1); JNK-associated membrane protein (JAMP); glucocerebrosidase (GCase); Erb-B2 receptor tyrosine kinase 3 (ErbB3).
